# Intense PSMA expression of the uvula detected by [^68^ Ga]Ga-PSMA-PET/CT

**DOI:** 10.1007/s00259-023-06511-x

**Published:** 2023-11-13

**Authors:** Lisa Glantschnig, Alexander Dierks, Georgine Wienand, Christian H. Pfob, Ralph A. Bundschuh, Constantin Lapa, Malte Kircher

**Affiliations:** Nuclear Medicine, Faculty of Medicine, University of Augsburg, Augsburg, Germany

With the increasing use of prostate-specific membrane antigen (PSMA) targeting positron emission tomography (PET) imaging for prostate cancer staging, different radioligand uptake patterns of normal organs, e.g., the kidney, small intestine, or salivary glands, have been detected. In particular, the latter is of special interest due to the toxicity of radioligand therapy with [^177^Lu]Lu-PSMA ligands potentially resulting in dose-limiting xerostomia [1].

Recently, the claimed discovery of an allegedly unknown pair of salivary glands aroused great public attention [2, 3]. By means of PSMA-PET/computed tomography (PET/CT), a group of Dutch researchers described a bilateral structure posterior in the nasopharynx, with ligand uptake similar to the known major salivary glands that they called “tubarial glands” [2]. In the aftermath, the novelty of the finding was severely questioned given the fact that glands in this region of the throat had already been described in the nineteenth century [4].

In our case, a 74-year-old man with newly diagnosed high-risk prostate cancer was referred for primary staging. Whole-body PSMA-PET/CT with [^68^ Ga]Ga-PSMA-I&T revealed the primary tumor but no extraprostatic focus. As an incidental finding, intense PSMA expression was detected in the uvula (SUV_max_ 6.71; red arrows). Anatomic studies have described the human uvula to consist of serous and seromucous glandular masses, muscular tissue, and large excretory canals. Thus, it is capable of producing large quantities of fluid saliva [5].

Noteworthy, the so-called “tubarial glands” could also be non-invasively visualized (SUV_max_ 7.44; blue arrows).

Although we cannot claim to have discovered a novel human organ, our case highlights the ability of PSMA-directed molecular imaging to detect salivary gland tissue within the human body. In addition—as demanded by Horace in his *Ars Poetica*—it could please and educate the reader and thus serve as an interesting piece of information.
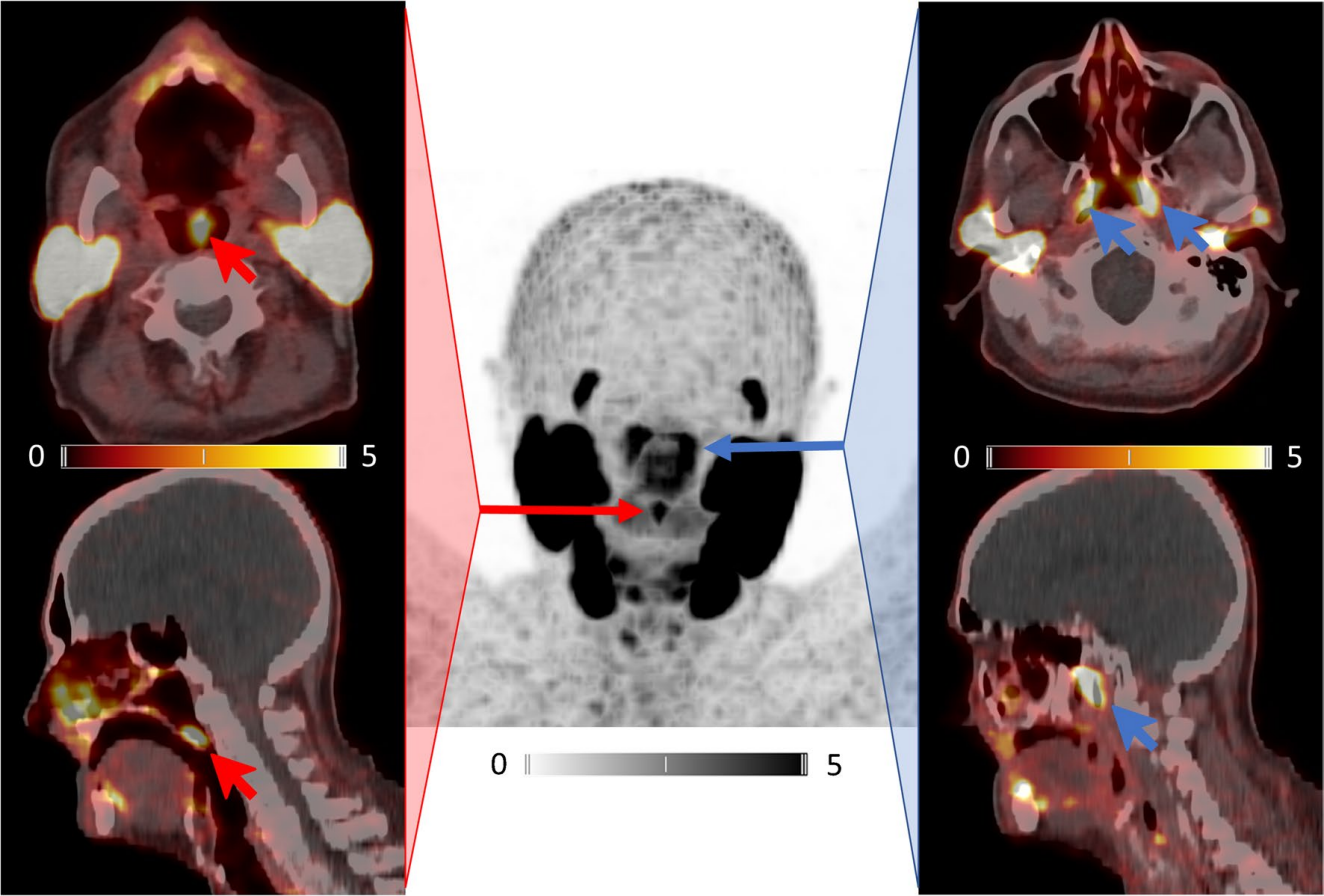


## Data Availability

The original dataset is available from the corresponding author upon reasonable request.

## References

[1] Heynickx N, Herrmann K, Vermeulen K, Baatout S, Aerts A (2021). The salivary glands as a dose limiting organ of PSMA- targeted radionuclide therapy: a review of the lessons learnt so far. Nucl Med Biol.

[2] Valstar MH, de Bakker BS, Steenbakkers R, de Jong KH, Smit LA, Klein Nulent TJW (2021). The tubarial salivary glands: a potential new organ at risk for radiotherapy. Radiother Oncol.

[3] Wu KJ (2020). Doctors may have found secretive new organs in the center of your head.

[4] Mudry A, Jackler RK (2021). Are “tubarial salivary glands” a previously unknown structure?. Radiother Oncol.

[5] Finkelstein Y, Meshorer A, Talmi YP, Zohar Y, Brenner J, Gal R (1992). The riddle of the uvula. Otolaryngol Head Neck Surg.

